# MicroRNA and Renal Allograft Monitoring

**DOI:** 10.5812/numonthly.12722

**Published:** 2013-06-12

**Authors:** Sepide Zununi, Mohammadreza Ardalan

**Affiliations:** 1 Faculty of Advanced Medical Sciences, Tabriz University of Medical Sciences, Tabriz, IR Iran; 2 Chronic Kidney Disease Research Center, Tabriz University of Medical Sciences, Tabriz, IR Iran

**Keywords:** MicroRNAs, Kidney Transplantation, Rejection, Fibrosis

Micro RNAs (MiRNAs) are small endogenous, regulatory RNAs comprising of about 19–25 noncoding nucleotides (1-3). Primary miRNAs (pri-miRNA), a capped and polyadenylated transcript are transcribed by RNA polymerase II and processed into precursor miRNAs (pre-miRNAs) in the nucleus by the microprocessor complex (DGCR8/Drosha). Using Exportin-5, pre-miRNAs are exported into the cytoplasm and are cleaved by Dicer to produce mature miRNAs. Mature miRNAs recognize their mRNAs targets and impose their negative regulation of protein synthesis by degradation of corresponding mRNA (1-3). These single-stranded RNAs have essential roles in numerous biological and pathological processes and their aberrant expression is associated with disease initiation and/or progression due to a severe disturbance of downstream gene networks and signaling cascades and protein synthesis. miRNAs have been involved in regulation of inflammation, innate and adaptive immunity, fibrosis and in signaling mechanisms implicated in allograft rejections. miRNAs are detectable in several biologic sources including peripheral blood mononuclear cell (PBMC), serum, tissue samples, urinary cell pellets and urine supernatant and many other body fluids (4).

Acute rejection is the result of an alloimmune response against the allograft and can be caused by either a cellular or humoral response. An acute cellular rejection normally occurs 5 to 7 days after transplant but can occur in an accelerated fashion or any time after transplantation. Chronic rejection is a progressive deterioration in renal dysfunction characterized clinically by a progressive increase in serum creatinine level, and histologically by tubular atrophy, and interstitial fibrosis. It is an almost universal finding that finally happens in all renal transplant recipients and most commonly have an immunological reason. Previously the terminology of chronic allograft nephropathy (CAN) was used to describe these changes The Banff classification system for renal allograft injury has recently adopted the term interstitial fibrosis and tubular atrophy (IFTA) to describe these changes (5).

Despite an improvement in renal allograft survival because of advances in immunosuppressive regimens a critical area is the need for sensitive, etiology-specific and noninvasive method for monitoring the function of the renal allograft. Because serum creatinine increment is a very late findings when some irreversible damages has happened on the other hand renal biopsy is an invasive procedure and it is difficult to implement it as a routine procedure for renal allograft monitoring (6).

Several studies support the idea of earlier diagnosis of acute and chronic rejection by miRNA measurement (Table 1). In an earlier report microarray study of renal allografts biopsy samples disclosed the up regulation of miR-320 and down regulation of, miR-324-3p among the 20 different miRNAs in patient with acute rejection (7). Anglicheau et al. study a panel of 17 different miRNAs in biopsy samples of patients with acute rejection and they found a 100% sensitivity and 95% specificity for miR142-5p over expression as a diagnosing biomarker of acute rejection (8). The study by Lorenzen et al. offered a new opportunity for earlier diagnosis of cellular rejection and monitoring renal allograft function using urine sample as a source of miRNA measurement. In their study miR-10a were upregulated and miR-10b and miR-210 were down regulated in urine sample of patient with acute T-cell mediated rejection (9). The results of other studies showed that miR-223 measurement in peripheral blood mononuclear cells (PBMCs) can have a promising potential, with specificity of 90% and sensitivity of 92%, for earlier diagnosing of acute rejection (AR) (10). One recent study indicates that miR-142-5p is a promising biomarker for long-term renal allograft monitoring and with its elevation we could detect chronic antibody mediated rejection (CAMR) in its earlier stages they used PBMC and biopsy samples for their measurements (11). In another very recent publication Wilflingseder et al. revealed the distinct miRNA signatures of biopsy samples in acute cellular and humoral rejection and delayed graft function. In this study up regulation of the following miRNA panel; miR-182, miR-155, miR-125a, miR-146b, was associated with antibody mediated rejection (12).

**Table 1.** Different Studies on miRNA in Renal Transplantation

Condition/Monitoring	miRNAs	Sample Size/Source	Control Group	Ref.
**Renal allograft status**	Up: miR-125a, miR-125a, miR-320, miR-381, miR-602, miR-628, miR-629, miR-658Down: miR-17-3p, miR-197, miR-324-3p, miR-326, miR-330, miR-346, miR-483, miR-516-5p, miR-524, miR-611, miR-654, miR-663	3AR, 3C/Biopsy	Healthy nontransplant recipient volunteers	Sui et al., 2008 (7)
**AR** **^a^**	Up: miR-142–5p, miR-142–3p, miR-155, miR-223, miR-146b, miR-146a and miR-342down: let-7c, miR-10a, miR-10b, miR-125a, miR-200a, miR-30a-3p, miR-30b, miR30c, miR30e-3p, miR-32	12AR, 21C/Biopsy	Normal allograft biopsies (grafts from living and deceased donors)	Anglicheau et al., 2009 (8)
**Acute T-Cell** **mediated rejection**	Up: miR-10aDown: miR-10b, miR-210	62AR, 19C, Urine samples	Transplant patients without rejection	Lorenzen et al., 2011 (9)
**AR**	Up:miR-223	12 AR, 21 C/PBMC	Normal controls	Liu et al., 2011 (10)
**Chronic antibody-mediated rejection**	Up: miR-301a, miR-590-5p, miR-142-5p, miR-32, miR-503 down: miR-888, miR-576-5p, miR-548b-5p, miR-125b, miR-194	112 patient (53 STA, 40 CAMR, 10 RF, 9 AR), 11 C/ PBMC and Biopsy	Healthy volunteers	Danger et al., 2013 (11)
**ABMR** **^a^** **, AREJ** **^a^** **, DGF** **^a^**	ABMRup: miR-146-5p, miR-1228, let-7i, miR-21, miR-182, miR-155, miR-125a, miR-146bAREJ Up: miR-150, miR-155, miR-663, miR-638Down: 18 miRNAs; miR-125b-2, mir-99b, mir-30c-2, mir-424, DGFUp: miR-182, miR-106b, miR-20a, miR-21, miR-18a, miR-17, miR-106a	55 patient [41AR (15 VR, 15 IR, and 11 ABMR), 14 DGF], 10C /Biopsy	Normal protocol biopsies	Wilflingseder et al., 2013 (12)
**AR**	miR-125b, miR-483, miR-663, miR-326, miR-346, miR-125a, miR-381, miR-602, miR-629, miR-324-3p, miR-658, miR-524	3 AR, 3 C/Biopsy	Normal kidney biopsies	Sui et al., 2013
**IF/TA** **^a^**	56 miRNAs differentially expressedUp: miR-142-3p, miR-32Down: miR-204, miR-107, miR-211	81 KTR tissue biopsy samples and urinary cell pellets (deceased donor kidneys)	Stable normal allografts	Scian et al., 2011 (13)
**IF/TA**	33 miRNAsUp: miR-21, 142-3p, and 5p and the cluster comprising miR-506Down: miR-30b and 30c	8 cases (4 IFTA and 4C) Validation set: 18 cases (10 IFTA and 8 C)/ Biopsy	Normal biopsies	Ben-Dov et al., 2012 (14)
**IF/TA**	50 differentially expressed miRNAs in UUO mouse modeUp: miR-21	42 renal transplanted recipients/serum		Glowacki et al., 2013

^a^Abbreviations: AR, acute rejection; ABMR, antibody-mediated rejection; AREJ, acute cellular rejection; CAMR, chronic antibody mediated rejection; DGF, delayed graft function; IF/TA, interstitial fibrosis and tubular atrophy; KTR, kidney transplant recipients; PBMC, peripheral blood mononuclear cell

The major hallmarks of progressive renal allograft dysfunction are interstitial fibrosis and tubular atrophy (IF/TA). It usually starts after the first transplantation year and results in a continuous decrease in renal allograft function. In the preliminary publication of miRNA profiling in CAD with IF/TA, 56 differential regulation of miRNAs were studied in tissue samples and urinary cell pellets. Up regulation of: miR-142-3p, miR-32 and down regulation of: miR-204, miR-107, miR-211 were observed in their studied population with IF/TA (13). All those five miRNAs, were detected from the urinary cell pellets of the histologically diagnosed patients (13). Ben-Dov and colleagues studied a panel of 33 different miRNAs in the biopsy samples of patients with IF/TA and compared it with normal biopsy samples. They found a higher expression of miR-21, 142-3p, and 5p and the cluster comprising miR-506 on chromosome X and lower expression of miR-30a, miR-30d and miR-30e in IF/TA biopsies (14). It has been reported that several miRNAs comprised of miR-21, miR-200 family (miR-200a, miR-200b, miR-200c, miR-429 and miR-141), miR-29 family (miR-29a, miR-29b-1, miR-29b-2 and miR-29c), miR-192, miR-217, miR-377, miR-93, miR-382 and miR-216 all are affected by TGF-β expression, a key fibrogenetic cytokine involved in fibrosis (1, 3). The prominent role of miR-21 (2, 15-18), miR-29 family (19-22) and miR-200 family (23, 24) in kidney transplant fibrosis have been pronounced. Recent study of Glowacki et al. (2013) suggested that miR-21 by itself is a novel, predictive and reliable blood marker of kidney allograft fibrosis (16).

In the context of kidney disease and early noninvasive diagnosis, urine is a specimen of choice. It provides a representative sampling of the entire kidney and an indirect way to visualize intragraft compartment; therefore, it is naturally appealing to nephrologists (25). The role of urinary miRNA as a feasible method is under active research for diagnosis of different disease such as chronic kidney disease (26, 27), diabetic nephropathy (28), adult nephrotic syndrome (29) and severity of fibrosis in Immunoglobulin A nephropathy IgA (22).

The recent discovery of human microRNAs (miRNA) opens a new horizon to biomedical research Several publications have supported the value of urinary miRNAs for diagnosis renal allograft dysfunction so it could be a useful tool to monitor the status of the kidney allograft function and a feasible biomarker of IF/TA (15).

## References

[1] Mas VR, Dumur CI, Scian MJ, Gehrau RC, Maluf DG (2013). MicroRNAs as biomarkers in solid organ transplantation.. Am J Transplant..

[2] Patel V, Noureddine L (2012). MicroRNAs and fibrosis.. Curr Opin Nephrol Hypertens..

[3] Scian MJ, Maluf DG, Mas VR (2013). MiRNAs in kidney transplantation: potential role as new biomarkers.. Expert Rev Mol Diagn..

[4] Weber JA, Baxter DH, Zhang S, Huang DY, Huang KH, Lee MJ (2010). The microRNA spectrum in 12 body fluids.. Clin Chem..

[5] Solez K, Colvin RB, Racusen LC, Haas M, Sis B, Mengel M (2008). Banff 07 classification of renal allograft pathology: updates and future directions.. Am J Transplant..

[6] Ling XB, Sigdel TK, Lau K, Ying L, Lau I, Schilling J (2010). Integrative urinary peptidomics in renal transplantation identifies biomarkers for acute rejection.. J Am Soc Nephrol..

[7] Sui W, Dai Y, Huang Y, Lan H, Yan Q, Huang H (2008). Microarray analysis of MicroRNA expression in acute rejection after renal transplantation.. Transpl Immunol..

[8] Anglicheau D, Sharma VK, Ding R, Hummel A, Snopkowski C, Dadhania D (2009). MicroRNA expression profiles predictive of human renal allograft status.. Proc Natl Acad Sci U S A..

[9] Lorenzen JM, Volkmann I, Fiedler J, Schmidt M, Scheffner I, Haller H (2011). Urinary miR-210 as a mediator of acute T-cell mediated rejection in renal allograft recipients.. Am J Transplant..

[10] Liu XY, Xu J (2011). [The role of miR-223 in the acute rejection after kidney transplantation].. Xi Bao Yu Fen Zi Mian Yi Xue Za Zhi..

[11] Danger R, Paul C, Giral M, Lavault A, Foucher Y, Degauque N (2013). Expression of miR-142-5p in peripheral blood mononuclear cells from renal transplant patients with chronic antibody-mediated rejection.. PLoS One..

[12] Wilflingseder J, Regele H, Perco P, Kainz A, Soleiman A, Muhlbacher F (2013). miRNA profiling discriminates types of rejection and injury in human renal allografts.. Transplantation..

[13] Scian MJ, Maluf DG, David KG, Archer KJ, Suh JL, Wolen AR (2011). MicroRNA profiles in allograft tissues and paired urines associate with chronic allograft dysfunction with IF/TA.. Am J Transplant..

[14] Ben-Dov IZ, Muthukumar T, Morozov P, Mueller FB, Tuschl T, Suthanthiran M (2012). MicroRNA sequence profiles of human kidney allografts with or without tubulointerstitial fibrosis.. Transplantation..

[15] Chau BN, Xin C, Hartner J, Ren S, Castano AP, Linn G (2012). MicroRNA-21 promotes fibrosis of the kidney by silencing metabolic pathways.. Sci Transl Med..

[16] Glowacki F, Savary G, Gnemmi V, Buob D, Van der Hauwaert C, Lo-Guidice JM (2013). Increased circulating miR-21 levels are associated with kidney fibrosis.. PLoS One..

[17] Wang J, Gao Y, Ma M, Li M, Zou D, Yang J (2013). Effect of miR-21 on Renal Fibrosis by Regulating MMP-9 and TIMP1 in kk-ay Diabetic Nephropathy Mice.. Cell Biochem Biophys..

[18] Zarjou A, Yang S, Abraham E, Agarwal A, Liu G (2011). Identification of a microRNA signature in renal fibrosis: role of miR-21.. Am J Physiol Renal Physiol..

[19] Fang Y, Liu Y, Kriegel AJ, Heng Y, Xu X, Liang M (2013). MiR-29c is down-regulated in renal interstitial fibrosis in humans and rats and restored by HIF-α activation.. Am J Physiol Renal Physiol..

[20] Qin W, Chung AC, Huang XR, Meng XM, Hui DS, Yu CM (2011). TGF-beta/Smad3 signaling promotes renal fibrosis by inhibiting miR-29.. J Am Soc Nephrol..

[21] Wang B, Komers R, Carew R, Winbanks CE, Xu B, Herman-Edelstein M (2012). Suppression of microRNA-29 expression by TGF-beta1 promotes collagen expression and renal fibrosis.. J Am Soc Nephrol..

[22] Wang G, Kwan BC, Lai FM, Chow KM, Li PK, Szeto CC (2012). Urinary miR-21, miR-29, and miR-93: novel biomarkers of fibrosis.. Am J Nephrol..

[23] Oba S, Kumano S, Suzuki E, Nishimatsu H, Takahashi M, Takamori H (2010). miR-200b precursor can ameliorate renal tubulointerstitial fibrosis.. PLoS One..

[24] Xiong M, Jiang L, Zhou Y, Qiu W, Fang L, Tan R (2012). The miR-200 family regulates TGF-beta1-induced renal tubular epithelial to mesenchymal transition through Smad pathway by targeting ZEB1 and ZEB2 expression.. Am J Physiol Renal Physiol..

[25] Anglicheau D, Suthanthiran M (2008). Noninvasive prediction of organ graft rejection and outcome using gene expression patterns.. Transplantation..

[26] Lorenzen JM, Haller H, Thum T (2011). MicroRNAs as mediators and therapeutic targets in chronic kidney disease.. Nat Rev Nephrol..

[27] Szeto CC, Ching-Ha KB, Ka-Bik L, Mac-Moune LF, Cheung-Lung CP, Gang W (2012). Micro-RNA expression in the urinary sediment of patients with chronic kidney diseases.. Dis Markers..

[28] Argyropoulos C, Wang K, McClarty S, Huang D, Bernardo J, Ellis D (2013). Urinary microRNA profiling in the nephropathy of type 1 diabetes.. PLoS One..

[29] Wang G, Kwan BC, Lai FM, Chow KM, Li PK, Szeto CC (2013). Urinary sediment miRNA levels in adult nephrotic syndrome.. Clin Chim Acta..

